# Surface acoustic wave-based generation and transfer of droplets onto wettable substrates†

**DOI:** 10.1039/d2ra04089a

**Published:** 2022-08-17

**Authors:** Krishnadas Narayanan Nampoothiri, Niladri Sekhar Satpathi, Ashis Kumar Sen

**Affiliations:** Fluid Systems Laboratory, Department of Mechanical Engineering, Indian Institute of Technology Madras Chennai-600036 India ashis@iitm.ac.in; Micro Nano Bio Fluidics Group, Indian Institute of Technology Madras Chennai-600036 India

## Abstract

Fluid manipulation using surface acoustic waves (SAW) has been utilized as a promising technique in the field of microfluidics due to its numerous advantages, over other active techniques, such as low power requirement, facile fabrication methods, and non-invasive nature. Even though SAW-based generation of micron-sized droplets through atomization has been studied, the role of substrate wettability on the characteristics of the transferred droplets has not been explored to date. Here, we study the generation and effective transfer of micron-sized droplets using SAW onto wettable substrates whose water contact angles vary from 5° to 145°. The characteristics of transferred droplets after impacting the wettable substrates are characterized in terms of the contact line diameter and polydispersity index. A theoretical model is formulated to predict the initial average size of the transferred droplets on the wettable substrates of different contact angles. The variation of polydispersity and number density with contact angle is explained by considering droplet coalescence and bouncing. The relevance of the technique in biological assays is demonstrated by transferring droplets of streptavidin protein samples onto a substrate.

## Abstract 2

Investigation of surface acoustic wave-based generation and transfer of droplets onto wettable substrates is carried out and explained in terms of polydispersity index. Transfer of biomolecules is demonstrated to highlight the technique.

## Graphical abstract

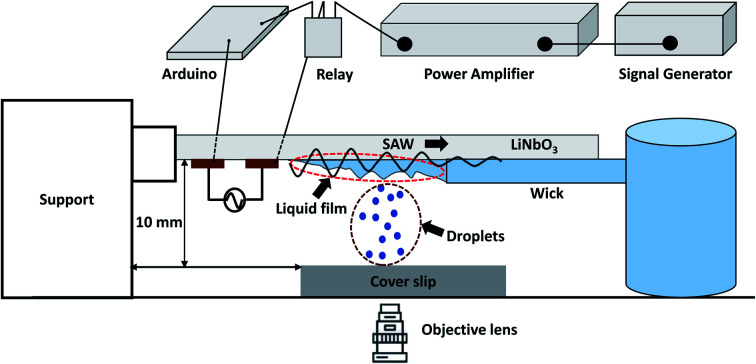

## Introduction

Manipulation of microliter-size droplets is of prime importance in the field of microfluidics^1–4^ given its plethora of applications in medical science,^5,6^ electronics cooling,^7,8^ printing,^9–11^ and aerosol formation.^12,13^ Active forces such as electric^14–16^ and magnetic field forces^17,18^ have been extensively used to handle small sample volumes. These techniques, however, involve complex fabrication,^19^ require high electric fields,^20,21^ and use complex reagents^22^ which adversely affect the versatility of such methods. The literature indicates an urgent need for an enhanced method for the efficient handling of small fluid volumes. Surface acoustic waves (SAW) based techniques can fill this gap given their low power requirements, simple and robust fabrication methods, and non-invasive characteristics. Techniques based on SAWs have proven competency^23,24^ for droplet manipulation,^25^ droplet atomization,^26–28^ and aggregation of particles.^29^ In particular, the non-contact nature of SAWs is well-disposed for biochemical applications.^30,31^

SAWs are defined as mechanical vibrations of nanometric amplitudes that propagate on the surface of a piezoelectric substrate.^23^ Upon applying an AC electrical signal to interdigitated electrodes (IDTs), mechanical traveling waves are formed on the surface of the piezoelectric substrate as a consequence of the inverse piezoelectric effect. When SAWs encounter a liquid, leaky SAWs are introduced into the bulk of the liquid at the Rayleigh angle.^22,32^ When sufficient power is applied, strong capillary waves at the air-droplet interface overcome the capillary stress and form micron-sized satellite droplets.^28,33–35^ The atomized droplet sizes are typically measured during their flight using time of flight experiments^28,36^ and using this mechanism, droplets of size in the range of 1 to 100 μm were generated in a rapid and controlled manner.^24,37^ The ease of mass-fabrication of SAW devices provides SAW atomizers^38,39^ with an edge over ultrasonic atomizers.^40,41^

From the application perspective, SAW-based atomization has been reported as an effective technique for extraction of bioreagents,^26^ transfer of analytes into a mass spectrometer for peptide detection,^35^ drug delivery,^42–44^ and the production of insulin liquid aerosols and solid protein nanoparticles.^39^ Qi *et al.*^26^ demonstrated the usage of paper strips for SAW atomization and extracted protein molecules and yeast cells (∼5 μm) from narrow paper strips. Sun *et al.*^35^ presented an integrated SAW-based chip that could control the nebulization rate of the non-volatile analytes during atomization. Huang *et al.*^42^ utilized the SAW-based nebulization for asthma treatment. Alhasan *et al.*^43^ utilized this SAW atomization platform for efficient pulmonary stem cell delivery. Rajapaksa *et al.*^44^ demonstrated the delivery of a pDNA vaccine in a large animal model using SAW based hand-held nebulizer. Owing to the scaled-down device and absence of nozzles, the SAW-based atomization technique can be utilized for cooling electronic devices.^45^ Huang *et al.*^46^ investigated the effect of acoustothermal heating and measured the temperature distribution of liquid atomization during SAW actuation. To avoid the effect of acoustothermal heating in efficient transfer of proteins and cells, literature suggests the usage of low SAW powers (<4 W).^23,47^ Even though the transport of droplet-encapsulated bioreagents has been demonstrated in the literature, the effect of substrate wettability on the characteristics of these transferred droplets has not been investigated. This study is critical for the transfer of monodisperse droplets which has relevance in various applications such as cosmetics,^48^ food,^49^ and chemical industries.^50^ These droplets can also serve as microvessels for carrying out various bio-detection techniques.^51,52^

In the present work, we have investigated the characteristics of the transferred droplets after impacting onto various wettable substrates. These droplets are generated using SAW technique on LiNbO_3_ substrate and the source liquid used is in the form of a thin liquid film which is proven to offer a higher atomization rate as compared with that obtained using microliter volume droplets.^28,32,53^ For such a study, surfaces of different wettability with water contact angles (WCA) in the range of 5° to 145° are prepared using a glass coverslip as the base substrate. The transferred droplets on the different wettable substrates are imaged to obtain the droplet characteristics such as the size distribution and the polydispersity index. A theoretical model is presented that predicts the average initial size of the transferred droplets on the wettable substrates. During these experiments, the wettability of the LiNbO_3_ substrate is unaltered since literature^54^ suggests that as the wettability of the LiNbO_3_ substrate is changed to hydrophobic/superhydrophobic the area fraction through which the surface acoustic waves leak into the droplet decreases and there will be no effect of surface acoustic waves. Finally, the potential application of the technique is demonstrated by transferring streptavidin protein molecules.

## Experimental section

**Fig. 1:**
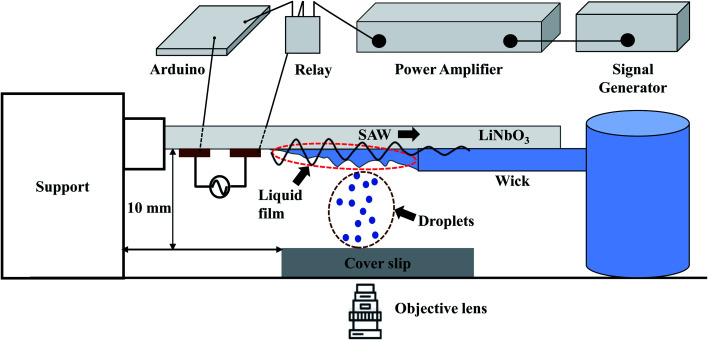
Schematic diagram of the experimental setup depicting the generation of liquid film, SAW-based droplet atomization, and transfer of atomized droplets to a substrate.

A schematic diagram of the experimental setup is depicted in Fig. 1. Interdigitated electrodes (IDTs) of Cr/Au of thickness 10/100 nm are fabricated on the surface of a 128° rotated Y-cut X propagating lithium niobate (LiNbO_3_) substrate (thickness 0.5 mm) using standard photolithography and metal etching techniques. The width and inter-electrode spacing are taken to be 100 μm, and the corresponding resonant frequency *f*_r_ is found to be 9.91 MHz. Traveling surface acoustic waves (TSAWs) are generated on the LiNbO_3_ substrate by applying sinusoidal AC voltage signals to the IDTs, which are produced using a signal generator (SMC100A, Rohde & Schwarz, Germany) and subsequently amplified by a power amplifier (75A250A, Amplifier Research). The gain of the power amplifier is set at 35.6 dB and the input power from the signal generator is kept at 0 dBm (1 mW). By considering the electromechanical coupling coefficient^55,56^ of 128° rotated Y-cut X propagating LiNbO_3_ as 5.5%, the RF power converted to the acoustic modes is 0.2 W. Mechanical relay switch (YL303H RS series) on an Arduino circuit works as a timer set at 100 ms to keep the SAW power ON for 100 ms after which the power is switched OFF for another 100 ms and the cycle continues.

A piece of polyester cellulose lint-free paper is used to wick the liquid from a reservoir through capillary action and to maintain a continuous liquid film front exposed to the SAWs.^32^ The surface of the liquid film undergoes capillary instability resulting in the ejection of small droplets that are deposited on surfaces of different wettability with a glass coverslip as the substrate, as shown in Fig. 1. The side view of the liquid film is captured using a high-speed camera (Photron Fastcam Mini AX200).

The temperatures of the substrates were measured using a thermal IR camera (A6701, FLIR). The IR camera is calibrated against an external thermometer by measuring the temperature of a water bath, which is heated using a hot plate.

Streptavidin (biotin-binding proteins) covalently attached to a fluorescent label, Qdot™ 625 Streptavidin conjugate, is transferred from the liquid film to the substrate. Optiprep™ (Sigma-Aldrich) is used as the density gradient medium to ensure Streptavidin particles remain neutrally buoyant.

The images of the transferred droplets and the biotin-binding proteins are captured using a 60× objective lens attached to an inverted fluorescent microscope (IX71, Olympus) in combination with a high-speed camera (SA5, Photron) and a fluorescence attachment (Olympus U-HGLGPS). The size distribution of the transferred droplets are analyzed using ImageJ software.^57^

**Fig. 2:**
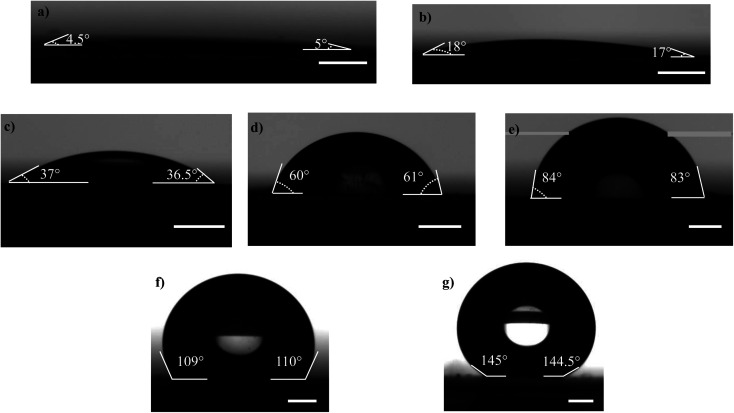
Water Contact Angles (WCAs) of the substrates after surface treatment of the coverslip by following different protocols (a) 5° (b) 18° (c) 37° (d) 60° (e) 84° (f) 110° and (g) 145°. Scale bar = 0.5 mm.

Microscopic coverslips (Blue Star, India) of thickness 0.17 mm are used as the substrates for transferring the atomized droplets (Fig. 1). The water contact angle of the coverslip substrate (WCA) is varied in the range of 5° to 145°, as shown in Fig. 2, by using the protocols described below. The various contact angles are measured using a Goniometer and Drop Shape Analyzer (DSA25, KRÜSS GmbH). Each measurement is repeated at least thrice on multiple substrates having the same wettability to ensure repeatability and reproducibility.

PDMS base and curing agent at 10 : 1 ratio are thoroughly mixed and the mixture is degassed in a desiccator to remove air bubbles. The coverslip substrate is washed with isopropyl alcohol in an ultrasonicator and dried by spraying high-pressure N_2_ gas. The PDMS mixture is spin-coated on a glass coverslip at 4000 rpm for 50 s and cured at 140 °C for 1 h resulting in a uniformly coated and cured PDMS layer. The PDMS surface is then exposed to oxygen plasma (PDC – 002, Harrick Plasma, U.S.A.) at 30 W for 90 s,^58^ and the WCA of the PDMS surface immediately after the exposure is measured to be 5° ± 0.2° (Fig. 2a). In addition to the wettability of the substrate, the surface roughness can also have an effect on the droplet distribution. PDMS substrate will have nanometric roughness, as reported in another study from our group.^59^ But here we keep the surface roughness fixed and only study the effect of wettability. The variation of the wettability of a plasma exposed PDMS surface with time has been investigated elsewhere,^60^ which is attributed to the adsorption of the –OH bonds into the PDMS layer.^60^ From our measurements, we found the WCA of PDMS surfaces to be 18° ± 0.1° after 8 h (Fig. 2b) and 37° ± 0.3° after 24 h (Fig. 2c). The WCA of the cleaned coverslip and PDMS surface without plasma exposure after curing for 140 °C for 1 h is measured to be 60° ± 0.2° and 84° ± 0.3°, respectively (Fig. 2d and e respectively), which are consistent with values reported in the literature.^61,62^

The obtained PDMS surface is considered rigid since the elastocapillary number length^63,64^ (*γ*/*E*) , where *γ* is the liquid surface tension and *E* is the Young's modulus of PDMS,^65^ is considered to be low (≈10^−8^ m). To achieve hydrophobic surface, the spin-coated PDMS layer is cured at 180 °C for 90 min,^66^ and the WCA is measured to be 110° ± 0.3° (Fig. 2f). To obtain a superhydrophobic surface, the glass coverslip is coated with 30 nm diameter silica nanoparticles (Glaco Mirror coat, Soft 99, Japan) and heated at 110 °C for 1 h to reinforce the coating.^55^ The coating offers a surface having micro and nanoscale dimensions and the WCA is measured to be 145° ± 0.2° (Fig. 2g). The substrate has very low (∼±2°) contact angle hysteresis which ensures the minimal effect of the same in spreading and retraction of the droplet upon impact. Since experiments with each of the wettable surfaces are completed within 15 min during which the WCA variation is insignificant,^60^ the effect of the dynamic wettability change on the droplet characteristics can be safely neglected. The different protocols used to achieve different WCAs are summarized in Table 1.

**Table 1:** The different protocols used to achieve different WCAs of the substrates

Substrate surface	Curing condition	Plasma exposure time	Waiting time	WCA
Glass coverslip	Not applicable	Not applicable	Not applicable	60° ± 0.2°
Spin coated PDMS	140 °C, 1 h			84° ± 0.3°
Spin coated PDMS	180 °C, 90 min			110° ± 0.3°
Silica nanoparticle coating	110 °C, 1 h			145° ± 0.2°
Plasma exposed PDMS	Not applicable	30 W for one and half min	Not applicable	5° ± 0.2°
			8 h	18° ± 0.1°
			24 h	37° ± 0.3°

## Results and discussion

### Formation of liquid film and transfer of droplets

**Fig. 3:**
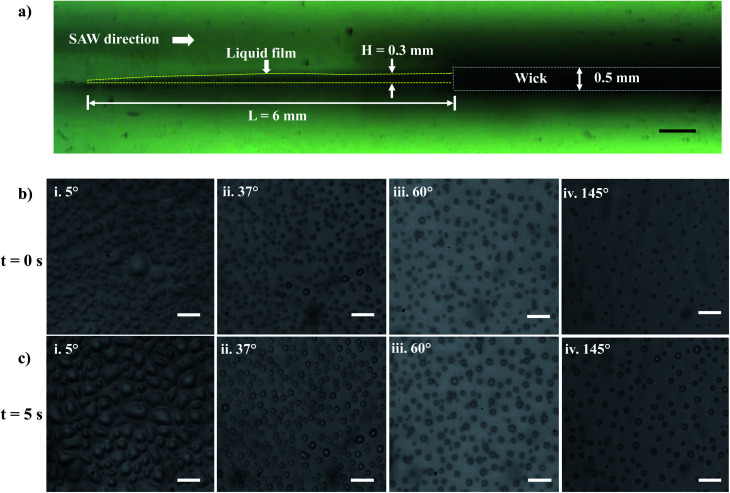
(a) Experimental image (side view) illustrating the wick and the liquid film characteristics during SAW actuation. Scale bar = 500 μm. (b) Experimental images of the droplets on surfaces having different WCA at *t* = 0 s: (b-i) 5°, (b-ii) 37°. (b-iii) 60°, (b-iv) 145°. (c) Experimental images of the droplets on surfaces having different WCA at *t* = 5 s: (c-i) 5°, (c-ii) 37°, (c-iii) 60°, (c-iv) 145°. Scale bar = 10 μm.

Experiments are performed to establish a liquid film over the LiNbO_3_ substrate for the SAW-mediated droplet generation, as shown in Fig. 3a. Lint-free paper of thickness 0.5 mm and 50 mm length is connected to a reservoir containing DI water at one of its ends, which resulted in the complete wetting of the paper within 60 s. The contact between the edge of the wetted paper wick at the other end and the hydrophilic LiNbO_3_ substrate led to the formation of a liquid meniscus of thickness 0.2 mm and length 0.5 mm. The coverslip substrate is placed at a vertical distance of 10 mm from the LiNbO_3_ substrate as shown in Fig. 1. When the liquid meniscus is exposed to SAW actuation with the timer circuit set at 100 ms, the meniscus spreads opposite to the direction of SAW propagation, and a liquid film of height *H* = 0.3 mm and length *L* = 6 mm is produced, as shown in Fig. 3a. Further, the capillary instability^28^ leads to the breakup of the liquid film producing droplets that are collected on different coverslips having WCA in the range 5° to 145°.

Since the experiments are performed for a duration of 5 s, there might be the effect of acoustothermal heating which can produce heating effect on the atomized droplets. Thus, the temperature distribution of the substrate (with and without thin liquid film) is measured using a thermal camera. The emissivities of LiNbO_3_ and water are given as 0.71 (ref. 67) and 0.96.^68^ Fig. S1a† shows the thermal IR image of the substrate (with liquid thin film) when SAW is actuated for 10 s. A region of interest (ROI) is created and the average temperature of ROI during SAW actuation is measured. Fig. S1b† provides information about the average temperature rise for the dry substrate and for the thin liquid film for the same ROI. The maximum temperature rise for time *t* = 5 s is observed to be 2.25 °C and 0.25 °C for the wet and dry substrates respectively. Thus, the effect of acoustothermal heating can be negated during the experiments.

Within 1.0 ms of SAW actuation, droplets are observed on the coverslip substrate indicating that the timescale of instability and subsequent atomization is smaller than 1.0 ms and hence the processes occur quite rapidly.^27,37^ The experimental images of the droplets collected on the various wettable surfaces at *t* = 0 s and *t* = 5 s are presented in Fig. 3b and c and S2.† Here, *t* = 0 s denotes the time instant at which the droplets were first observed on the coverslip substrate, and *t* = 5 s represents the time instant just after 5 s. When the droplets impact the wettable coverslip substrates, depending on the WCA and the time elapsed, the contact line diameter of the drops *D*_m_ and the droplet number density (*N*_d_) on the coverslip will be different.

**Fig. 4:**
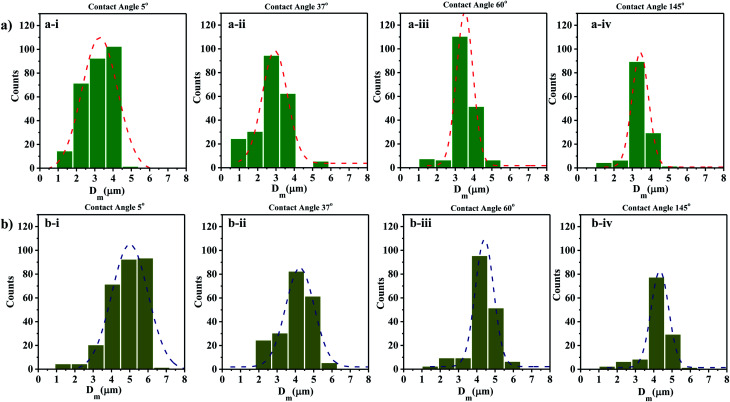
Droplet size distributions at (a) *t* = 0 s and (b) *t* = 5 s on surfaces having different WCA (a-i and b-i) 5° (a-ii and b-ii) 37° (a-iii and b-iii) 60° (a-iv and b-iv) 145°.

The size distribution of contact line diameters of the droplets at *t* = 0 s and *t* = 5 s on different substrates having different WCA are presented in Fig. 4a and b and S3.† From the captured images, ROI is chosen which could accommodate the maximum number of droplets. For consistency the coordinates of the ROI is kept constant for all images. The edges of the transferred droplets are detected using ImageJ through edge detection process.

It is observed that for smaller WCA (5°, 18°, and 37°), the droplet diameters fall in the range of 2 to 4 μm and 3 to 5 μm at *t* = 0 s and *t* = 5 s respectively. With an increase in the WCA between 60° and 145°, the droplet diameters are in the range of 3 to 5 μm and 4 to 6 μm at *t* = 0 s and *t* = 5 s respectively. This is quite non-intuitive as the contact line diameter of droplets is expected to be larger on surfaces having a smaller WCA. The results suggest that droplets impact on the surface at a time point earlier than one second, and smaller impacting droplets can stay over surfaces having smaller WCA. On the other hand, smaller droplets impacting over surfaces having higher WCA roll-off or bounce back and only larger droplets formed through coalescence with incoming droplets stay on such surfaces. For all the surfaces, a polydisperse nature is observed in the size distribution of the droplets. Depending on the contact angle of the wettable substrate where these droplets are allowed to impact, only some of the droplets are captured. As seen in Fig. 4a and b, an increase in the diameter range between *t* = 0 s and *t* = 5 s is due to the coalescence of the droplets on the surfaces. The evaporation of thin films (1–50 μl) is reported to be in minutes.^69^ Since the SAW actuation and subsequent atomization effects are performed within a few seconds, the effects of evaporation on the droplet transfer can be neglected. The effect of evaporation on the size of the transferred droplets is not considered here.

### Theoretical model – prediction of contact line diameter

**Fig. 5:**
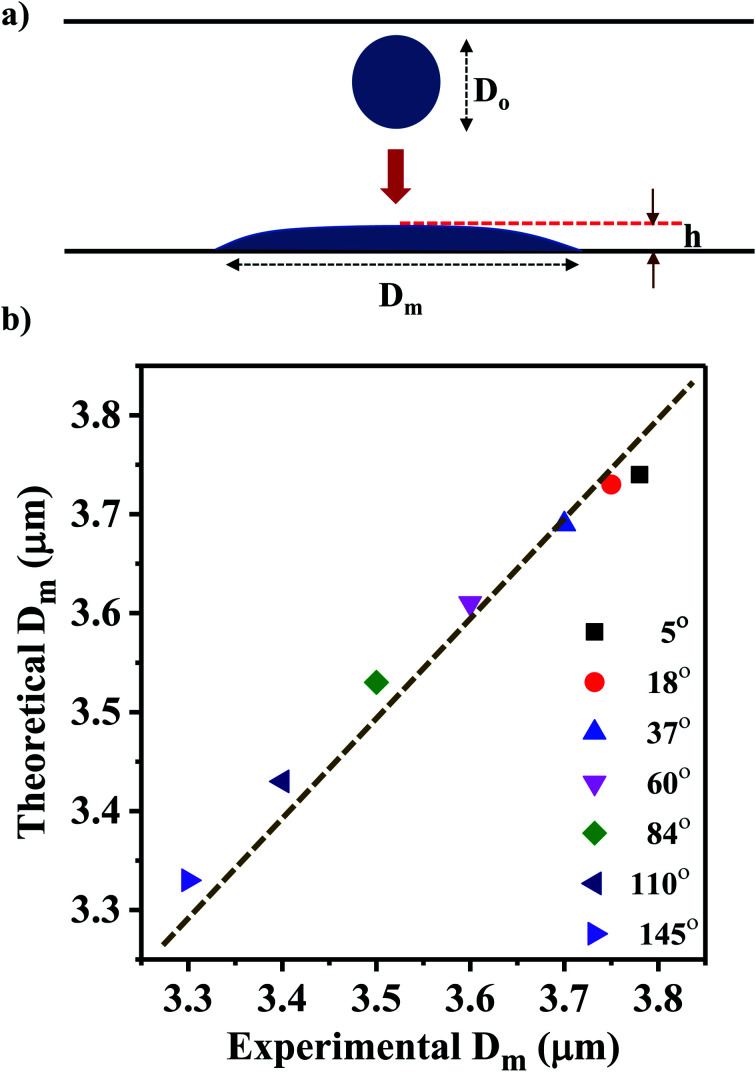
(a) Schematic diagram of the model geometry depicting a droplet before and after impact. (b) Comparison of the theoretical *D*_m_ values with the measured experimental average *D*_m_ values.

We present a theoretical model by taking into account the various energies acting on a drop approaching the surface before impacting and during its spreading after impacting the surface. A schematic diagram of the model geometry depicting a droplet before and after impact is shown in Fig. 5a. Here, ‘*h*’ represents the splat thickness at the maximum spread position, and *D*_o_ is the diameter of an ejected droplet that is estimated by characterizing the liquid film that is formed due to wicking.

Considering *H* and *L* as the characteristic height and length scale of the liquid thin film, which is generated during SAW actuation, Wang *et al.*^36^ reported an expression of the diameter of ejected droplets (*D*_o_) as follows,1
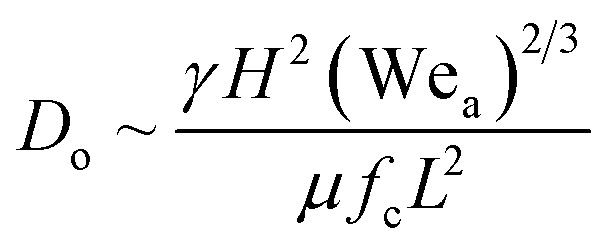
where *γ* and *μ* represents the surface tension and the dynamic viscosity of the liquid, respectively, *f*_c_ is the frequency of the surface waves and We_a_ is the acoustic Weber number. We_a_ is defined as^32^2
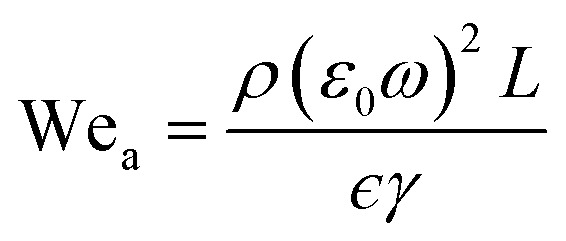
where *ρ* is the liquid density, *γ* is the liquid surface tension and *ω* = 2*πf*_r_ is the SAW angular frequency. *ϵ* is taken as *H*/*L* and *ε*_0_ is the SAW vertical displacement amplitude which is approximated as 2.8 nm from the following equation^55,70^3
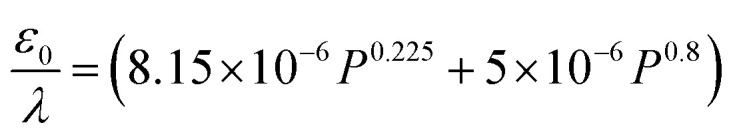
where *P* is the RF power and *λ* is the wavelength. By placing the values in eqn (2), We_a_ was calculated to be 52.

Depending on the geometric profile of the generated liquid film, *i.e.* (*ϵ* = *H*/*L*) ratio, different expressions are suggested for estimating the *f*_c_. For *ϵ* ≪ 1, the regime is considered to be a capillary resonant condition due to the internal viscous damping of the liquid and *f*_c_ is estimated as4
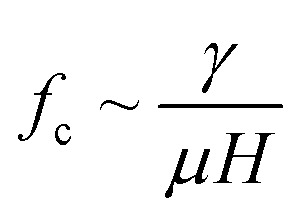


On the other hand, for *ϵ* ≫ 1, the regime is considered to be capillary resonant frequency due to the inertial forcing of the drop and *f*_c_ is estimated as5
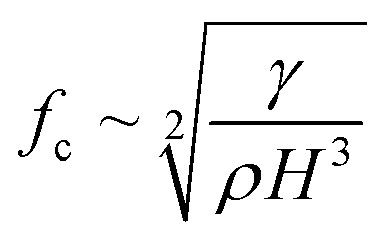


In the present case, since *ϵ* ≪ 1, as seen in Fig. 3a, and the regime can be considered satisfying the viscous-capillary resonant condition^28,32,36^ and the capillary wave frequency can be calculated from eqn (4) as *f*_c_ ∼ 0.27 MHz. Thus, by taking the different values into eqn (1), *D*_o_ is calculated as 3.08 μm.

The ejected droplets from the liquid meniscus impact the surface and undergo spreading to attain their equilibrium contact line diameters at the maximum spreading position. A general approach is to balance the sum of the initial kinetic energy (KE_i_) and interfacial energy (SE_i_) of the ejected droplets with the sum of the viscous energy dissipated during spreading (VE) and interfacial energy at the maximum spread (IE_m_) position on the substrate.^71–73^ Hence, from the principle of conservation of energy we get,

6 KE_i_ + SE_i_ = VE + IE_m_

where,7
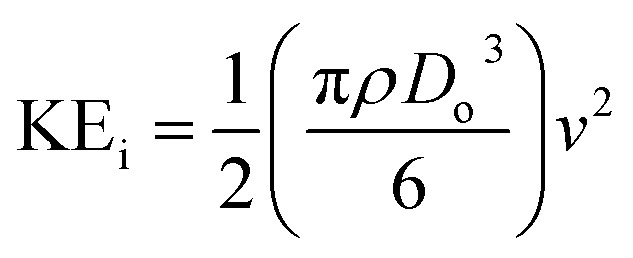

8 SE_i_ = π*γD*_o_^2^

9
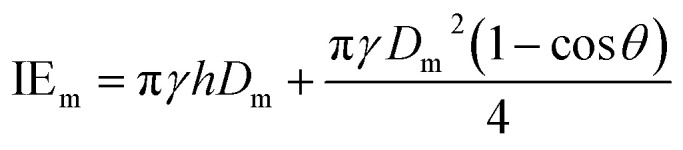


Here *v* represents the velocity of the droplet just before impacting on the surface and *θ* represents the WCA. Normally, *h* is calculated^73^ by equating the volume of a spherical droplet of diameter *D*_o_ with that of a cylinder of height *h* and diameter *D*_m_ as the splat is assumed to be cylindrical in shape as follows,10
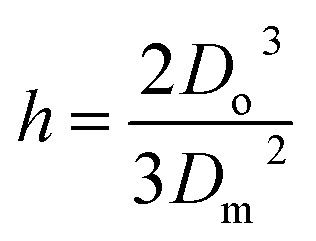


Now, VE is estimated from the energy spent to overcome the friction force,^73,74^ which is given as follows,11
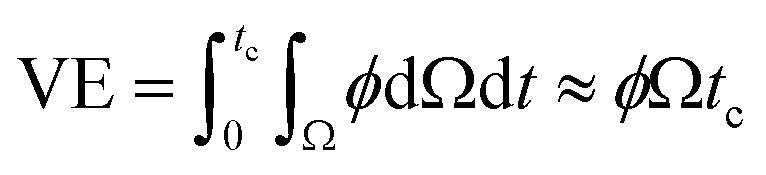
where 
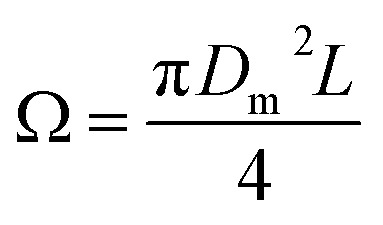
 is the volume of the boundary layer at the bottom of the droplet where viscous losses are significant, *t*_c_ is a phenomenological time, taken as the time taken for the droplet to spread completely, and *ϕ* ∼ *μ*(*v*/*L*)^2^ is the viscous dissipation function.^73–75^ Here, 
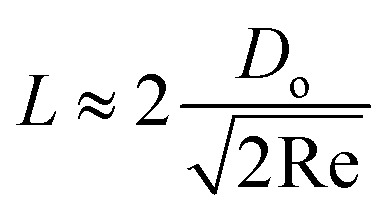
 denotes the characteristic length in the vertical direction,^72^*t*_c_ can be estimated^73^ by assuming the drop spreads to a diameter *D*_m_ and height *h*. By integrating for the evolution of *D*_m_, the phenomenological time is expressed as 
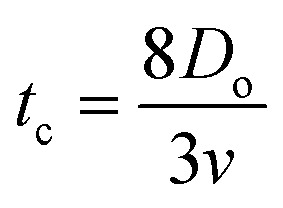
.

Upon substitution of the above parameters in eqn (11), VE is expressed as12
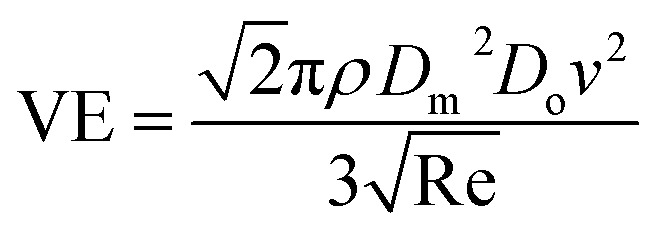


Combining the eqn (7)–(9) and (12) and substituting in eqn (6), and taking the dimensionless maximum spreading parameter as 
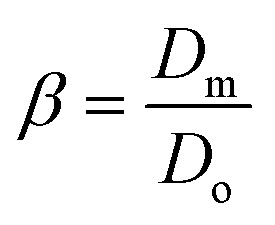
, we get13
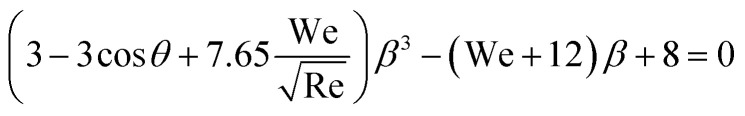
where the dimensionless numbers We and Re are expressed as 
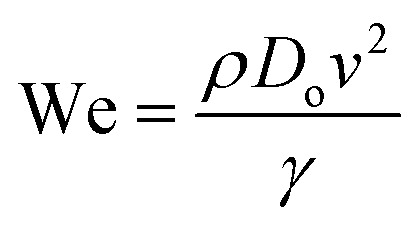
 and 
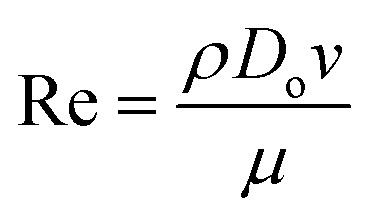
.

To obtain *β*, it is essential to know the velocity *v* of the droplet during the impact. Due to experimental constraints in measuring individual droplets during their impact on various wettable substrates, the approximate value of the velocity is taken into consideration. Through experimental observations, the time droplets taken to appear on the coverslip substrate, after SAW actuation, is measured as 0.6 ms. From literature, the time for the mist generation is known to be 0.2 ms,^27,37^ thus the time of flight is found to be 0.4 ms. The coverslip substrate is kept at a distance of 10 mm from the LiNbO_3_ substrate, using this the velocity of the drop just before impact is estimated to be *v* ≈ 25 m s^−1^. By placing the values in eqn (13), *β* is calculated and *D*_m_ is obtained by taking *D*_o_ as 3.08 μm.

This model predicts the initial droplet spreading diameter when the time *t* = 0 s and does not take into consideration the impact of multiple droplets. Thus, the obtained theoretical values is compared with the experimental average *D*_m_ for various WCAs which is measured at time *t* = 0 s. The experimental average *D*_m_ is calculated from the Gaussian size distribution (Fig. 4a).

Based on the size distribution for the droplets captured, the experimental average *D*_m_ for various WCAs is measured and compared with the theoretical results obtained from eqn (13) (Fig. 5b). The results show that the *D*_m_ decreases with the increase in WCA experimentally and the same trend is observed for the theoretical values also. Even though the error between experimental and theoretical values is minimal (<1%), there is scope of improvement for the theoretical model if the velocity of the drop can be measured accurately rather than estimating the magnitude of the velocity.

### Droplet polydispersity, number density, and average inter-distance

As evident from the experimental images, the size distribution of the droplets deposited on the substrate is nonuniform, which can be characterized using the polydispersity index (PDI). The polydispersity index (PDI) is defined^76^ as the square of the ratio of standard deviation (*σ*) and mean (*d*) of the droplet sizes and is mathematically defined as, PDI = (*σ*/*d*)^2^.

**Fig. 6:**
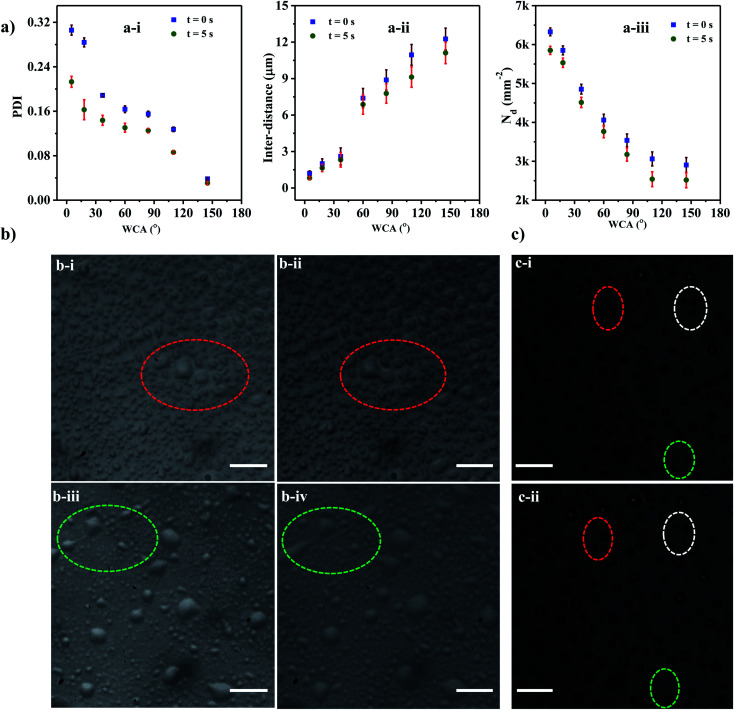
(a) Plots depicting (a-i) polydispersity (a-ii) average inter-distance with time (a-iii) droplet density (b) optical images showing the dynamic nature of the transferred droplets (b-i) at *t* = 0 s (WCA = 5°), (b-ii) at *t* = 0.5 s (WCA = 5°), (b-iii) at *t* = 0 s (WCA = 18°) and (b-iv) at *t* = 0.5 s (WCA = 18°). Scale bar = 10 μm. (c) Optical images showing the dynamic nature of the transferred droplets (c-i) at *t* = 0 s (WCA = 145°) and (c-ii) at *t* = 0.5 s (WCA = 145°). Scale bar = 10 μm.

The variation of PDI with WCA at time *t* = 0 s and 5 s are presented in Fig. 6a(a-i). It is observed that the PDI decreases with an increase in the WCA, which can be attributed to the fact that droplets deposited onto a hydrophilic surface tend to spread more and therefore have a higher tendency to coalesce leading to a higher PDI. For a given surface with a fixed WCA, the successive coalescence of droplets gives rise to the formation of a larger number of uniform coalesced droplets, leading to a reduction in polydispersity with time, as observed between *t* = 0 and 5 s. The experimental images of coalescence of adjacent droplets on surfaces having WCA = 5° and 18° are depicted in Fig. 6b(b-i–iv).

We find that the PDI is dependent on the interdistance between the adjacent droplets, which in turn is related to *N*_d_. The interdistance between adjacent droplets is smaller for a surface with a smaller WCA (Fig. 6a(a-ii)). As WCA increases the droplet spreads less on the surface and the tendency of the droplet to rebound and leave the surface increases hence a lower droplet density is observed with a higher WCA (Fig. 6a(a-iii)). On the other hand, at lower WCA, the droplets tend to spread more thereby maximizing their *D*_m_^71,77^ and incurring higher viscous losses which reduce their tendency to rebound from the surface and also reduce the inter-distance between the droplets. In the case of the superhydrophobic surface with WCA of 110° and 145°, PDI is found to be less than 0.1 for all times indicating a mono-disperse nature.^78,79^ PDI is found to be >0.1 for all other cases indicating the polydisperse nature of the formed droplets.

On superhydrophobic surfaces with WCA of 110° and 145°, the droplets tend to rebound from the surface^77,80^ and some disappear quickly after getting deposited onto the surface. In Fig. 6c(c-i and ii) the droplets encircled with dotted lines disappear after 0.5 s. With the reduction in droplet size, the capillary forces become stronger leading to a higher rebounding tendency than larger droplets.^72^ Further, for smaller size droplets, the surface area to volume ratio is higher leading to a higher rate of evaporation. As some of the smaller droplets rebound off the surface or quickly evaporate, the average inter-distance increases thereby reducing the coalescence tendency. This leads to a reduction in the droplet density thus providing a monodisperse droplet distribution at higher WCAs.

### Deposition of biomolecules on a surface – application of SAW-based droplet transfer

The capability of the droplet transfer technique for the transfer of biomolecules from a liquid sample onto the surface is demonstrated by deposition of biomolecules onto a wettable substrate. For such a study, it is necessary to choose a substrate with an appropriate WCA. The droplets carrying biomolecules should be deposited on the substrate rather than being repelled away. Therefore, substrates whose WCAs are hydrophobic and superhydrophobic are not considered. Even though the droplets stick to the surface for superhydrophilic surfaces, the tendency of merging (interdistance < 6 μm as shown in Fig. 6(a-ii)) with other droplets lead to large PDI nature (>0.16 as shown in Fig. 6(a-ii)). Thus, we chose coverslip as the substrate whose WCA is 60°.

**Fig. 7:**
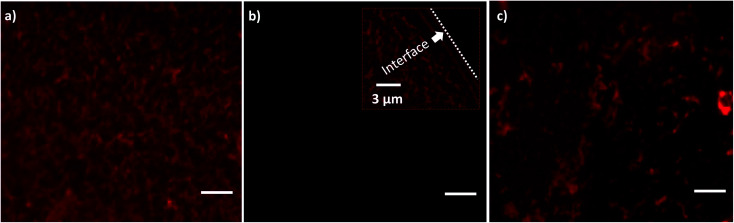
Fluorescent images showing (a) the presence of proteins on SAW substrate before actuation. (b) Absence of proteins on coverslip substrate before transfer (inset shows the transferred droplet with proteins). (c) Presence of proteins on the coverslip substrate after droplet evaporation. Scale bar = 10 μm.

A liquid sample (10 μl) containing quantum dot conjugated streptavidin solution and 10% Optiprep is prepared and loaded onto the lint-free paper. After drying the paper strip at room temperature, it is soaked with DI water from the reservoir through the wicking process and a liquid meniscus containing protein molecules is established. A fluorescent image of the proteins in the liquid meniscus is shown in Fig. 7a. Initially, before SAW actuation, the coverslip substrate is devoid of any protein (Fig. 7b). When SAW is actuated with the timer circuit set at 100 ms, droplets containing the proteins get transferred (inset of Fig. 7b) onto the coverslip substrate. The interface in the inset of Fig. 7b demarcates the regions of the substrate deposited with droplets containing protein molecules and the bare substrate. An experimental image of the proteins transferred onto the substrate, after complete evaporation of the droplets, is presented in Fig. 7c. While transferring the droplets onto the wettable surface by SAW, the coffee ring effect was not observed on the LiNbO_3_ substrate. Suppression of the coffee ring effect by acoustic waves has been reported in the literature.^81^ The dynamic patterns of solute particle formed inside the liquid film, during SAW actuation, inhibit the evaporation driven transport of the particles towards the contact line, thereby suppressing the coffee-ring effect. Thus, the low-power non-contact method of producing droplets from a liquid meniscus can be used for the transfer of bioreagents onto preferred substrates.

## Conclusion

This manuscript demonstrates the generation and transfer of micron-sized droplets onto varying wettable substrates using SAW-based actuation. Thin liquid film produced over a hydrophilic LiNbO_3_ substrate using the wicking technique enables the generation of atomized droplets and thus the size distribution of contact line diameters of the droplets on different substrates having different WCA is studied. The results show that the droplet size distribution is smaller in the case of a substrate with a smaller WCA, suggesting that smaller impacting droplets can stay over surfaces having smaller WCA but roll-off or bounce back over surfaces having higher WCA. A theoretical model is reported that predicts the average size of the transferred droplets on the substrates of different WCA, in good agreement with the experimental measurements. The atomized droplets are transported onto surfaces of different wettability and their characteristics such as polydispersity, and number density are investigated. Our study showed while the polydispersity decreases with an increase in WCA, but at the same time, the droplet–droplet interdistance increases or the droplet number density decreases for a higher WCA. So, depending on the application requirement, one can select a substrate of appropriate WCA. As the WCA of the substrate increases, the coalescence between adjacent droplets during its transfer reduces which leads to an increase in the interdistance between the droplets and a smaller polydispersity. The droplet number density also decreases due to evaporation and rebounding of the droplets from surfaces of higher WCA. The SAW-based droplet atomization technique is used to demonstrate the transfer of proteins from the liquid meniscus onto a substrate, highlighting its relevance in biological assays.

## Conflicts of interest

There are no conflicts to declare.

## Supplementary Material

RA-012-D2RA04089A-s001
