# Supplementary material for: Surface acoustic wave-based generation and transfer of droplets onto wettable substrates
Source: RSC Adv. 2022 Aug 17;12(36):23400–10. doi: 10.1039/d2ra04089a (PMC9382648; doi:10.1039/d2ra04089a)
Supplement: RA-012-D2RA04089A-s001 [file RA-012-D2RA04089A-s001.pdf]

Electronic Supplementary Material (ESI) for RSC Advances.
This journal is © The Royal Society of Chemistry 2022

SUPPLEMENTARY

Surface acoustic wave-based generation and transfer of droplets onto wettable substrates

Krishnadas Narayanan Nampoothiri[1,2], Niladri Sekhar Satpathi[1] and Ashis Kumar Sen[1,3*]

[1]Fluid Systems Laboratory, Department of Mechanical Engineering, Indian Institute of Technology Madras, Chennai-600036, India.

[2] Current Affiliation: Department of Mechanical Engineering, Amrita School of Engineering, Amrita Vishwa Vidyapeetham, Chennai-601103, India

[3]Micro Nano Bio Fluidics Group, Indian Institute of Technology Madras, Chennai-600036, India.

[*]Corresponding Author: ashis@iitm.ac.in

Thermal Images:

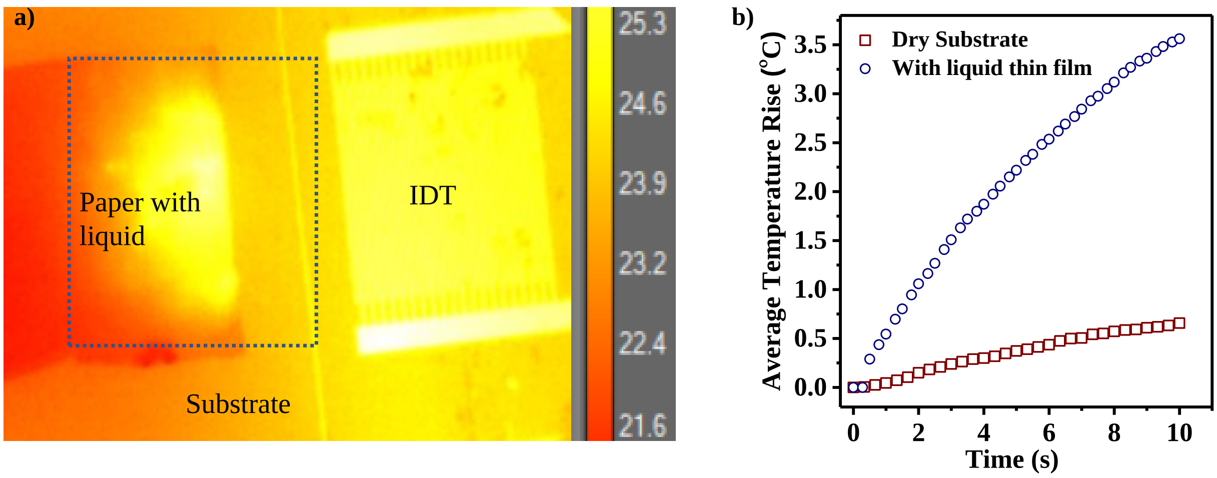

Fig. S1 (a) Thermal IR image of the substrate during SAW actuation at time t = 10 s. The temperature values in the thermal palette is in °C (b) Average temperature rise of the substrates (dry and wet) when SAW is actuated for 10 s.

Experimental images of the droplets on surfaces having different WCAs:

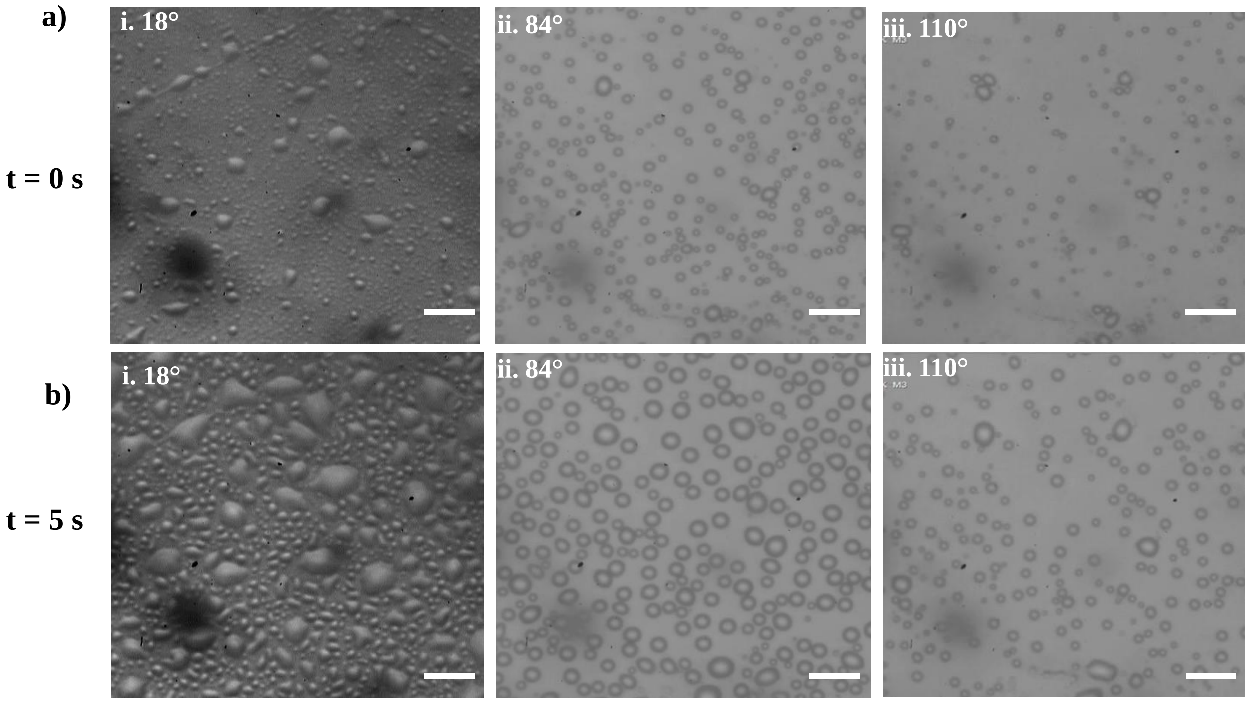

Fig. S2 (a) Experimental images of the droplets on surfaces having different WCA at t = 0 s: (a-i) 18°, (a-ii) 84°. (a-iii) 110°. (b) Experimental images of the droplets on surfaces having different WCA at t = 5 s: (b-i) 18°, (b-ii) 84°, (b-iii) 110°. Scale bar = 10 μm.

Droplet size distributions:

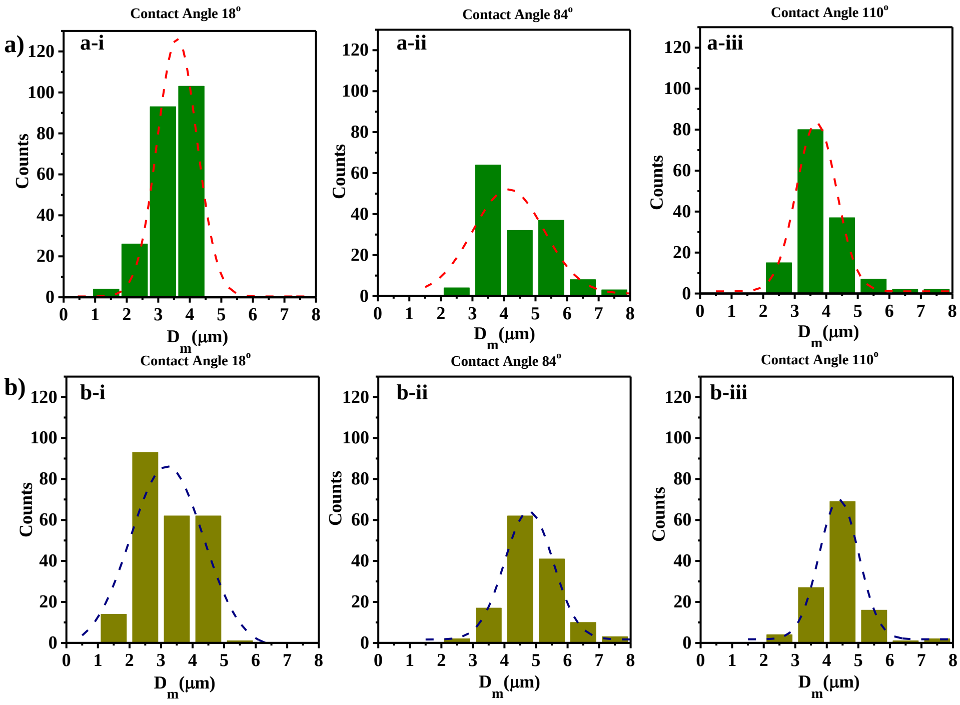

Fig. S3 Droplet size distributions at (a) t = 0 s and (b) t = 5 s on surfaces having different WCA (a-i and b-i) 18° (a-ii and b-ii) 84° (a-iii and b-iii) 110°.
